# Systematic Analysis of Transcriptomic Profile of Renal Cell Carcinoma under Long-Term Hypoxia Using Next-Generation Sequencing and Bioinformatics

**DOI:** 10.3390/ijms18122657

**Published:** 2017-12-07

**Authors:** Szu-Chia Chen, Feng-Wei Chen, Ya-Ling Hsu, Po-Lin Kuo

**Affiliations:** 1 Graduate Institute of Clinical Medicine, College of Medicine, Kaohsiung Medical University, Kaohsiung 807, Taiwan; scarchenone@yahoo.com.tw (S.-C.C.); s821721@hotmail.com (F.-W.C.); 2 Division of Nephrology, Department of Internal Medicine, Kaohsiung Medical University Hospital, Kaohsiung Medical University, Kaohsiung 807, Taiwan; 3 Department of Internal Medicine, Kaohsiung Municipal Hsiao-Kang Hospital, Kaohsiung Medical University, Kaohsiung 812, Taiwan; 4 Faculty of Medicine, College of Medicine, Kaohsiung Medical University, Kaohsiung 807, Taiwan; 5 Graduate Institute of Medicine, College of Medicine, Kaohsiung Medical University, Kaohsiung 807, Taiwan; yainghsu@kmu.edu.tw; 6 Institute of Medical Science and Technology, National Sun Yat-Sen University, Kaohsiung 804, Taiwan

**Keywords:** clear cell renal cell carcinoma, long-term hypoxia, next-generation sequencing, poor survival

## Abstract

Patients with clear cell renal cell carcinoma (ccRCC) are often diagnosed with both von Hippel-Lindau (VHL) mutations and the constitutive activation of hypoxia-inducible factor-dependent signaling. In this study, we investigated the effects of long-term hypoxia in 786-O, a VHL-defective renal cell carcinoma cell line, to identify potential genes and microRNAs associated with tumor malignancy. The transcriptomic profiles of 786-O under normoxia, short-term hypoxia and long-term hypoxia were analyzed using next-generation sequencing. The results showed that long-term hypoxia promoted the ability of colony formation and transwell migration compared to normoxia. In addition, the differentially expressed genes induced by long-term hypoxia were involved in various biological processes including cell proliferation, the tumor necrosis factor signaling pathway, basal cell carcinoma and cancer pathways. The upregulated (*L1CAM* and *FBN1*) and downregulated (*AUTS2*, *MAPT*, *AGT* and *USH1C*) genes in 786-O under long-term hypoxia were also observed in clinical ccRCC samples along with malignant grade. The expressions of these genes were significantly correlated with survival outcomes in patients with renal cancer. We also found that long-term hypoxia in 786-O resulted in decreased expressions of hsa-mir-*100* and hsa-mir-*378* and this effect was also observed in samples of metastatic ccRCC compared to samples of non-metastatic ccRCC. These findings may provide a new direction for the study of potential molecular mechanisms associated with the progression of ccRCC.

## 1. Introduction

Renal cell carcinoma (RCC) includes various malignancies that originate in the epithelium of renal tubules [1]. It is the most common form of adult kidney cancer, accounting for approximately 90–95% of cases. RCC can be classified into several histopathological types, of which clear cell RCC (ccRCC) is the most common. Partial or complete removal of the affected kidneys has been reported to be the initial treatment for ccRCC in 75–80% of cases [2,3]. Most cases of ccRCC are sporadic [4] and inherited ccRCC accounts for only 1–4% of cases [5]. Surgery is the treatment of choice for localized RCC, however metastasis develops in 20–30% of patients with limited disease at the time of surgery. The 5-year survival rate in patients with advanced RCC is low and the reported time to relapse after nephrectomy ranges from 15 to 18 months [6]. The mechanism of the development of RCC is unclear. The risk factors for RCC have been reported to be associated with carcinogens, chronic kidney inflammation and genetic alterations [7], however further studies are needed to elucidate the etiology.

The progression of cancer is a long and complicated process and often involves a microenvironment surrounding the tumor cells which is deficient of nutrients and oxygen. Tumor cells that adapt to a stressful environment can develop into a more aggressive phenotype and this almost always leads to metastatic disease [8]. One such critical stressful factor is hypoxia. Hypoxia-activated signaling occurs via the hypoxia-inducible factor (HIF)-1 signaling pathway, which regulates various molecules involved in proliferation, angiogenesis, migration and apoptosis [9,10]. Hypoxia has also been reported to affect chromosomal instability, leading to a disordered genetic expression [11,12]. The proteolytic regulation of HIF-α subunits is regulated by von Hippel-Lindau (VHL) protein, which is a ubiquitin ligase complex [13]. VHL-defective tumor cells have been shown to express high levels of HIF-α subunits (HIF-1α and HIF-2α), leading to the constitutive activation of hypoxia signaling [14,15]. VHL mutations in ccRCC have been found in sporadic (46–82%) and inherited cases [13].

There are two main types of hypoxia in human tumors according to traditional classification systems: long-term (4 h to several weeks) and short-term (30 min to 72 h) [16]. As both short- and long-term hypoxia represent different pathophysiologies with various biological and therapeutic consequences, distinguishing between them is important [16]. The main cause of long-term hypoxia is a severe restriction of oxygen diffusion from the microvessels of the tumor into the surrounding tissue. Cells receiving long-term hypoxia treatment for several weeks have been shown to have a higher invasive capacity than those receiving short-term hypoxia treatment [17]. However, few studies have evaluated the effects of long-term hypoxia in renal cancer. Recent evidence suggests that HIF-1α is mostly involved in short-term hypoxic responses, whereas HIF-2α is mostly involved in long-term hypoxic responses [18]. Therefore, in this study, we investigated the effects of long-term hypoxia (3 months) in a VHL-defective ccRCC cell line. We used 786-O, a VHL-defective ccRCC cell line lacking HIF-1α due to sequence depletion and constitutively expressing HIF-2α [13]. We hypothesized that cells under long-term hypoxia would become more aggressive. In addition, we hypothesized that long-term hypoxia may partially mimic a stressful microenvironment where tumor cells could grow and that the mechanisms may also be observed in clinical specimens with different grades of malignancy. There are currently no specific biomarkers for the prognosis or target therapy of ccRCC. Therefore, the aim of this study was to identify the novel genes involved in RCC progression and malignancy.

## 2. Results

### 2.1. Long-Term Hypoxia Promoted Colony Formation Ability and Transwell Migration Ability in the Von Hippel-Lindau (VHL)-Defective Clear Cell Renal Cell Carcinoma (ccRCC) Cell Line 786-O

**Figure 1:**
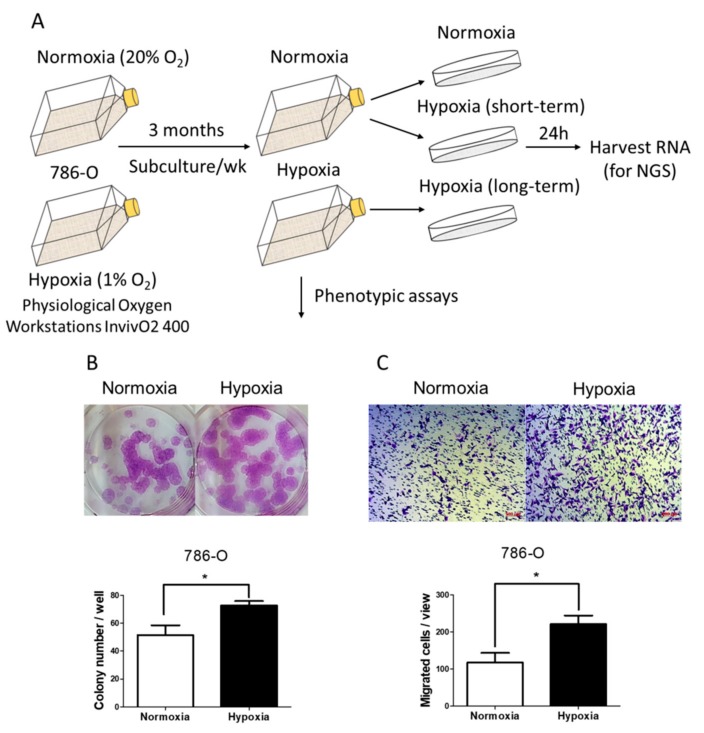
Long-term effects of hypoxia promoted colony formation ability and transwell migration ability in 786-O. (**A**) 786-O cells with the same number of passages were grown in normoxic and hypoxic conditions for 3 months. The normoxic conditions were 37 °C, 20% O_2_ and 5% CO_2_ and the hypoxic conditions were 37 °C, 1% O_2_ and 5% CO_2_. The long-term hypoxia-incubated 786-O cells were maintained in a physiological oxygen workstations InvivO_2_ 400 system. The cells were passaged once per week. Short-term hypoxia was performed with 24-h incubation. Total RNA lysates of the 786-O cells with the same number of passages under normoxia, short-term hypoxia and long-term hypoxia were harvested for next-generation sequencing (NGS); Colony forming ability (**B**) and transwell migration ability (**C**) were analyzed in the 786-O cells (with the same number of passages) maintained in either normoxic or hypoxic conditions for at least 3 months. (Scale bar = 100 μm) Each experiment was repeated at least three times. (*p*-values were calculated using the unpaired *t*-test, * *p*-value < 0.05).

We wanted to investigate the long-term effects of hypoxia in ccRCC using 786-O, a well-known VHL-defective ccRCC cell line lacking HIF-1α. We postulated that 786-O cells grown in long-term hypoxic conditions would develop more malignant characteristics. Our approach was to simultaneously maintain 786-O cells in normoxic and hypoxic conditions for at least 3 months. To avoid exposing the hypoxia-incubated cells to a normoxic environment during the subculture procedure, we maintained the cells in a pysiological oxygen workstations InvivO_2_ 400 system so that the subculture could be performed in hypoxic conditions. Both normoxic and hypoxic cells were passaged once every week on the same day to ensure that passage was equal. After 3 months, total RNA lysates were harvested for next-generation sequencing (NGS) and the cells were used in phenotypic assay analysis. To exclude short-term hypoxic effects, hypoxia was carried out for 24 h (Figure 1A). In the phenotypic analysis, the 786-O cells grown in long-term hypoxic conditions exhibited increased colony formation and transwell migration ability compared to normoxia (Figure 1B,C), indicating that long-term hypoxia promoted 786-O to become more aggressive.

### 2.2. Long-Term Hypoxia Altered the Expression Levels of Genes Involved in Various Biological Functions

**Figure 2:**
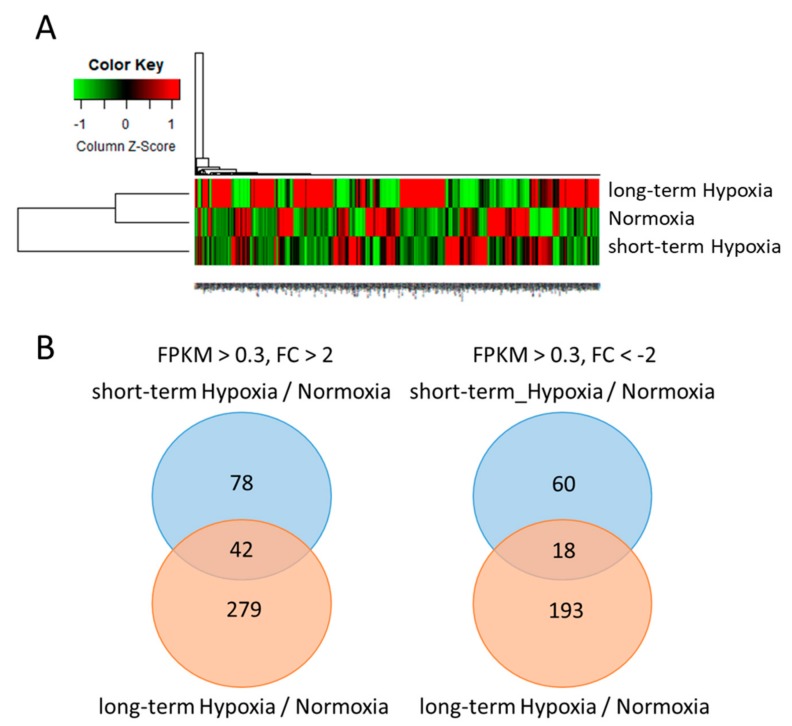
Analysis of protein-coding mRNA profiles using next-generation sequencing in 786-O cells under short- and long-term hypoxia. (**A**) A hierarchical clustering heat map showed the differentially expressed mRNAs in the 786-O cells that underwent normoxia, short-term hypoxia and long-term hypoxia. Scale = column Zscore (fragments per kilobase of transcript per million mapped reads, FPKM). The red and green colors represent higher and lower expression levels, respectively; (**B**) Venn diagram analysis showed 279 upregulated genes and 193 downregulated genes in 786-O cells that underwent long-term hypoxia compared to normoxia and 78 upregulated genes and 60 downregulated genes in 786-O cells that underwent short-term hypoxia compared to normoxia. There were 42 upregulated genes and 18 downregulated genes in both short- and long-term hypoxia. The criteria were a |fold change (FC)| >2 (short- or long-term hypoxia/normoxia) and fragments per kilobase of transcript per million mapped reads (FPKM) >0.3.

The sequencing data of 786-O cells grown in normoxic, short-term hypoxic and long-term hypoxic conditions were systematically analyzed and focused on protein-coding mRNAs and microRNAs. The threshold of protein-coding mRNAs was determined as fragments per kilobase of transcript per million mapped reads (FPKM) >0.3 and |fold change (long-term hypoxia/normoxia or short-term hypoxia/normoxia)| >2 (Figure 2A). We identified 279 upregulated genes and 193 downregulated genes in long-term hypoxia compared to normoxia and 78 upregulated genes and 60 downregulated genes in short-term hypoxia compared to normoxia (Figure 2B). 42 upregulated genes and 18 downregulated genes were observed in both short-term and long-term hypoxia, implying that these genes may be specifically regulated by hypoxia conditions in 786-O cells.

We used the Database for Annotation, Visualization and Integrated Discovery (DAVID) database for Kyoto Encyclopedia of Genes and Genomes (KEGG) pathway analysis [19]. In short-term hypoxia, the differentially expressed genes were significantly involved in the tumor necrosis factor (TNF) signaling pathway. In long-term hypoxia, five biological pathways were highly affected including basal cell carcinoma, TNF signaling pathway, cancer pathways, cancer proteoglycans and glycerolipid metabolism (Table 1), all of which have been reported to be associated with tumor progression-related functions.

**Figure 3:**
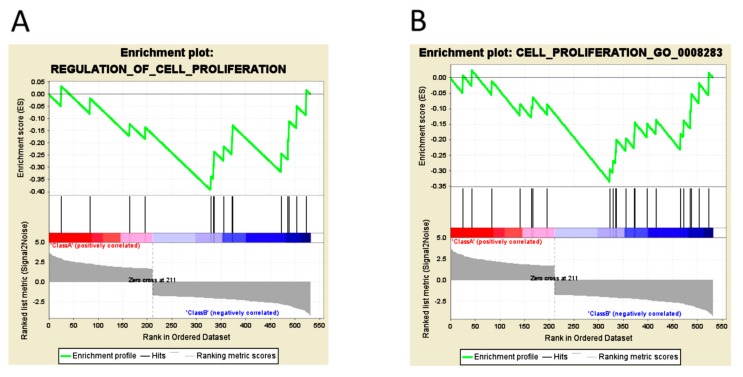
Gene set enrichment analysis (GSEA) of differentially expressed genes in 786-O with long-term hypoxia compared to normoxia. Gene set enrichment analysis (GSEA) was performed against differentially expressed genes in 786-O cells that underwent both long-term hypoxia and normoxia. (**A**,**B**) Two biological processes of cell proliferation were observed with strong correlations in long-term hypoxia. Class A (red) is normoxia group and class B (blue) is long-term hypoxia group. The detailed information is shown in Table 2.

In Gene Set Enrichment Analysis (GSEA), the biological processes were compared between normoxia (Figure 3A; Table 2) and long-term hypoxia (Figure 3B) and cell proliferation were upregulated in long-term hypoxia. These results suggested that the increased colony formation and transwell migration ability in 786-O cells under long-term hypoxia may be regulated via activation of various signaling pathways.

### 2.3. Identification of Potential Genes Associated with Advanced Malignancy of RCC Progression

**Figure 4:**
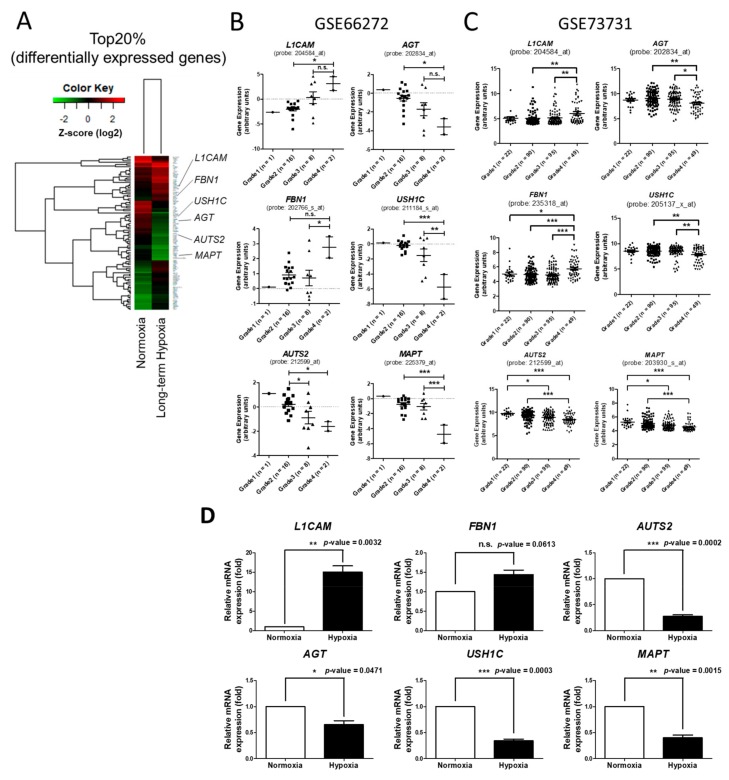
Analysis of the gene expression in clinical ccRCC samples with different malignant grades. (**A**) A hierarchical clustering heat map showing the top 20% of ranked genes in the 786-O cells with long-term hypoxia compared to normoxia; (**B**,**C**) The correlation of gene expression and ccRCC tumor grade was analyzed using microarrays (GSE66272 and GSE73731) data from the Gene Expression Omnibus (GEO) database. GSE66272 provides information of 27 clinical ccRCC samples (grade 1, *n* = 1; grade 2, *n* = 16; grade 3, *n* = 8; and grade 4, *n* = 2). GSE73731 provides information of 256 clinical ccRCC samples (grade 1, *n* = 22; grade 2, *n* = 90; grade 3, *n* = 95; and grade 4, *n* = 49); (**D**) qRT-PCR analysis of gene expression in 786-O cells treated with normoxia and long-term hypoxia. *GAPDH* was used as internal control. The raw data of GEO microarray datasets were obtained using the GEO2R online tool (Available online: https://www.ncbi.nlm.nih.gov/geo/geo2r/; accessed on 08 August 2017 and 23 November 2017) and re-plotted. (*p*-values of GEO microarray data were calculated using one-way ANOVA, qRT-PCR data were calculated using paired *t*-test, *** *p*-value < 0.001, ** *p*-value < 0.01, * *p*-value < 0.05 and n.s. = no significance).

We postulated that some differentially expressed genes identified in 786-O under long-term hypoxia may play important roles in the malignancy of clinical tumor progression. To identify potential targets, we searched microarrays (GSE66272 and GSE73731) using the Gene Expression Omnibus (GEO) database for advanced analysis. GSE66272 provides 27 clinical samples of ccRCC with grade 1 (*n* = 1), grade 2 (*n* = 16), grade 3 (*n* = 8) and grade 4 (*n* = 2). GSE73731 provides 256 clinical samples of ccRCC with grade 1 (*n* = 22), grade 2 (*n* = 90), grade 3 (*n* = 95) and grade 4 (*n* = 49). We excluded genes overlapping with short-term hypoxia and analyzed the top 20% of 279 upregulated genes and 193 downregulated genes in long-term hypoxia (Figure 4A). We found that the expression levels of two upregulated genes (*L1CAM* and *FBN1*) were also increased along with tumor grade and that the expression levels of four downregulated genes (*AUTS2*, *MAPT*, *AGT* and *USH1C*) were decreased along with tumor grade (Figure 4B,C). We next validated the expression of these genes in 786-O treated with normoxia and long-term hypoxia. The results showed that *L1CAM* was significantly upregulated and *AUTS2*, *MAPT*, *AGT* and *USH1C* were significantly downregulated in long-term hypoxia-treated 786-O (Figure 4D). *FBN1* was also observed with increased expression in long-term hypoxia-treated 786-O but there was no significant change (*p* = 0.0613). These findings suggested that the expression of these genes may play important roles in malignant progression of ccRCC.

**Figure 5:**
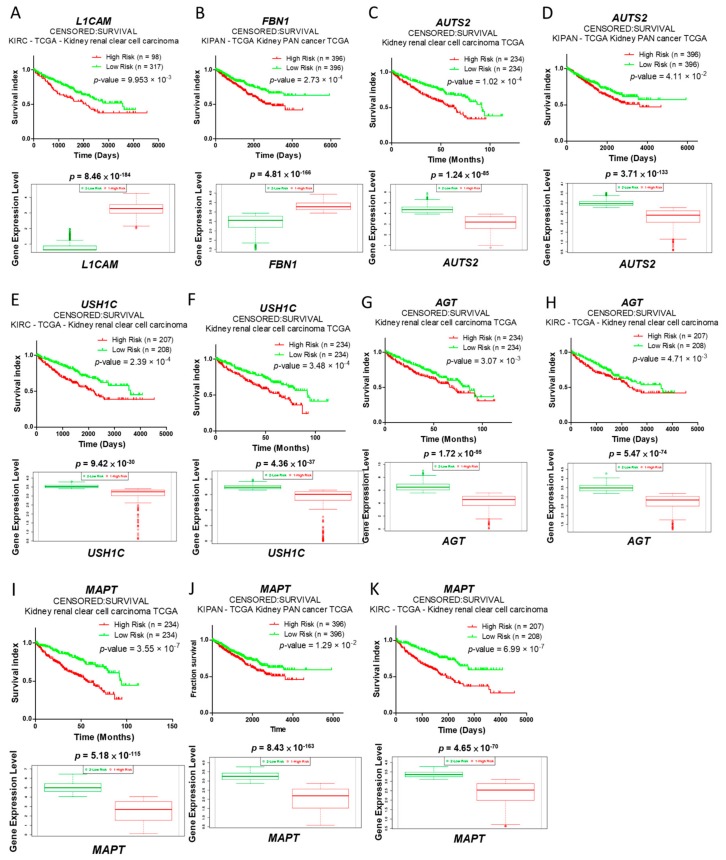
Correlation analysis of gene expressions and survival outcomes in renal cancer. The associations between survival outcomes and gene expressions of *L1CAM* (**A**); *FBN1* (**B**); *AUTS2* (**C**,**D**); *USH1C* (**E**,**F**); *AGT* (**G**,**H**); and *MAPT* (**I**–**K**) were analyzed using the SurvExpress database. TCGA-kidney cancer datasets were used for analysis. Red and green lines represent high- and low-risk groups, respectively. The box plot shows each gene expression in two groups (high and low risk).

We then used the SurvExpress database to analyze the expression effects of these seven candidates genes with regards to survival outcomes in patients with renal cancer. The results showed that a higher mRNA expression of either *L1CAM* or *FBN1* was associated with a poor survival outcome and that a lower mRNA expression of either *AUTS2*, *MAPT*, *AGT*, or *USH1C* was also associated with a poor survival outcome (Figure 5). These data suggest that during tumor progression, *L1CAM* and *FBN1* may exert a potentially oncogenic effect and that *AUTS2*, *MAPT*, *AGT* and *USH1C* may have potential tumor suppressor functions.

### 2.4. Analysis of Differentially Expressed microRNAs in 786-O under Long-Term Hypoxia 

**Figure 6:**
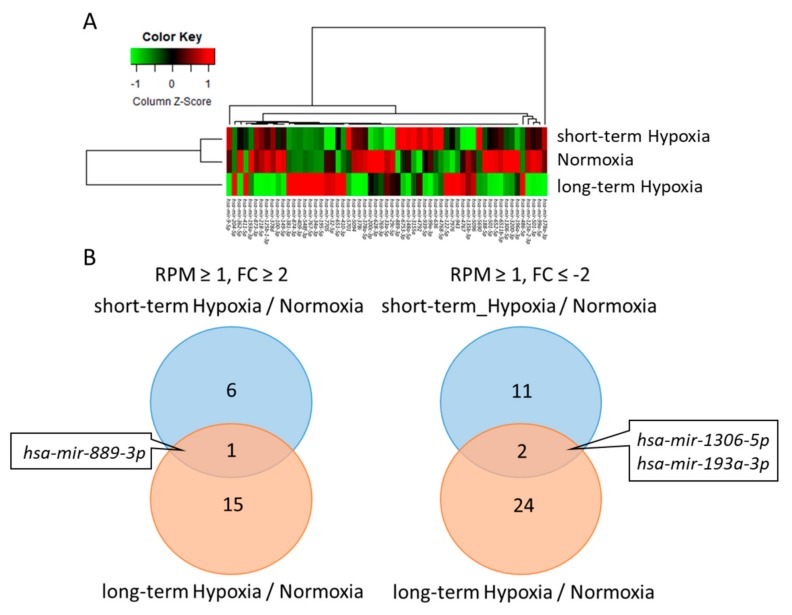
Analysis of microRNA profiles in 786-O with short- and long-term hypoxia compared to normoxia using next-generation sequencing. (**A**) A hierarchical clustering heat map showing the differentially expressed microRNAs in 786-O cells that underwent normoxia, short-term hypoxia and long-term hypoxia. Scale = column Z-score (reads per million, RPM). The red and green colors represent higher and lower expression levels, respectively; (**B**) Venn diagram analysis showed 15 upregulated genes and 24 downregulated genes in the 786-O cells undergoing long-term hypoxia compared to normoxia and 6 upregulated genes and 11 downregulated genes in 786-O cells undergoing short-term hypoxia compared to normoxia. There were 1 upregulated gene and 2 downregulated genes in both short- and long-term hypoxia. The criteria were a |fold change| ≥2 (short- or long-term hypoxia/normoxia) and reads per million (RPM) >1.

In addition to protein-coding mRNA, we also performed small RNA-seq to screen the microRNAs profile and to identify potential microRNA-mRNA interactions in 786-O cells under long-term hypoxia. Using NGS, the threshold was determined as reads per million (RPM) ≥1 and |fold change| ≥2 (Figure 6A). We identified 15 upregulated microRNAs and 24 downregulated microRNAs in long-term hypoxia and 6 upregulated microRNAs and 11 downregulated microRNAs in short-term hypoxia (Figure 6B). One microRNA (*hsa-mir-889-3p*) was upregulated and two microRNAs (*hsa-mir-1306-5p, hsa-mir-193a-3p*) were downregulated in both short-term and long-term hypoxia.

**Figure 7:**
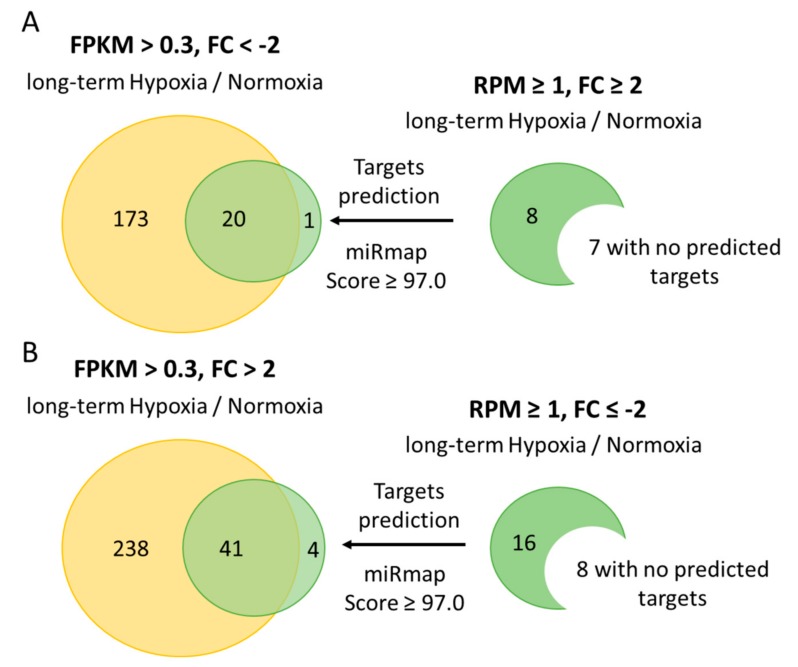
Analysis of putative microRNA-gene interactions using a target prediction database. The putative targets of differentially expressed microRNAs (excluding microRNAs overlapping in short-term hypoxia) in the 786-O cells undergoing long-term hypoxia were analyzed using the miRmap database. The criterion was defined as a miRmap score ≥97.0. Venn diagram analysis showed that (**A**) 20 putative targets of 8 upregulated microRNAs were also observed in the gene list of 193 downregulated mRNAs; and (**B**) 41 putative targets of 16 downregulated microRNAs were also observed in the gene list of 279 upregulated mRNAs.

To further identify putative target genes of microRNAs, we used the miRmap database (Available online: http://mirmap.ezlab.org/) for analysis with the criterion defined as a miRmap score ≥97.0. There were 21 putative targets of eight upregulated microRNAs (seven upregulated microRNAs with no predicted targets) and 45 putative targets of 16 downregulated microRNAs (eight downregulated microRNAs with no predicted targets) (Figure 7).

To identify potential microRNA-mRNA interactions, we observed whether the putative target genes of the upregulated microRNAs were downregulated in our mRNA sequencing data and vice versa. The results showed that 20 targets of upregulated microRNAs were also observed in the downregulated mRNA list and that 41 targets of downregulated microRNAs were observed in the upregulated mRNA list. TargetScan (Available online: http://www.targetscan.org/vert_71/) and miRDB (Available online: http://mirdb.org/) were used for second step target prediction (Table 3 and Table 4).

**Figure 8:**
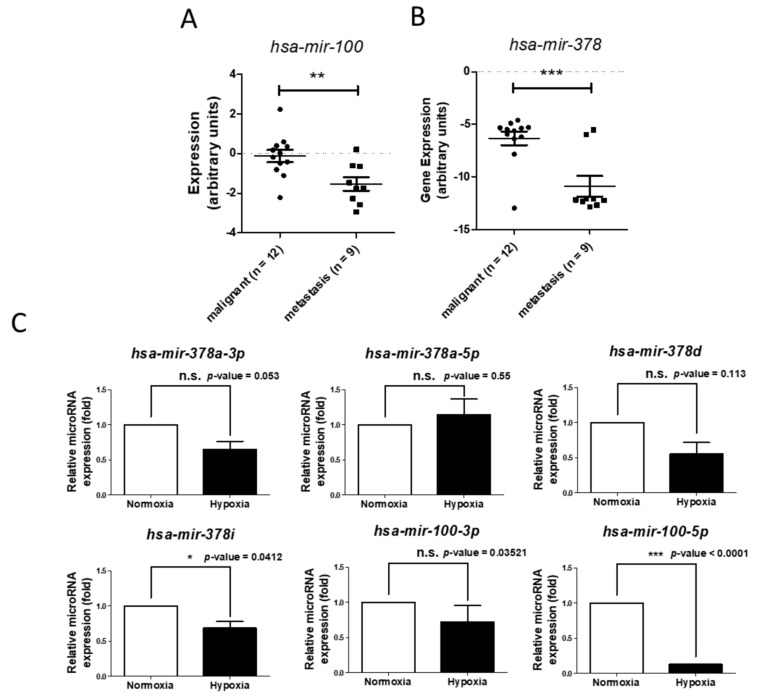
The expression levels of differentially expressed microRNAs, hsa-mir-100 (**A**) and hsa-mir-378 (**B**); identified in 786-O cells with long-term hypoxia compared to normoxia were analyzed on a microarray (GSE37989), which provides 21 clinical ccRCC samples (malignant ccRCC, *n* = 12; bone metastasis ccRCC, *n* = 9). The expression values were obtained using the GEO2R web tool and re-plotted; (**C**) qRT-PCR analysis of microRNAs expressions (hsa-mir-378a-3p, hsa-mir-378a-5p, hsa-mir-378d, hsa-mir-378i, hsa-mir-100-3p and hsa-mir-100-5p) in 786-O treated with normoxia and long-term hypoxia. U6 was used as internal control. (*p*-value of GSE37989 microarray and qRT-PCR were calculated by unpaired *t*-test and paired *t*-test respectively, * *p*-value < 0.05, ** *p*-value < 0.01, *** *p*-value < 0.001 and n.s. = no significance).

We next investigated whether the expression levels of microRNAs identified in 786-O cells under long-term hypoxia could be observed in clinical specimens along with malignancy. In microarray (GSE37989) which provided 12 clinical ccRCC and nine bone metastasis ccRCC specimens, the results showed that *hsa-mir-100* and *hsa-mir-378* are significantly decreased in bone metastatic ccRCC compared to malignant ccRCC (Figure 8A,B). In qRT-PCR analysis, we found that *hsa-mir-100-5p* and *hsa-mir-378i* were significantly decreased in long-term hypoxia treated 786-O (Figure 8C), suggesting the potential functions of *hsa-mir-100* and *hsa-mir-378* in renal cancer progression.

In this study, we maintained the VHL-defective ccRCC cell line under long-term hypoxic conditions to partially mimic a stressful environment where tumor cells develop in the human body. Transcriptomic profile analysis showed that two genes (*L1CAM* and *FBN1*) were upregulated and that four genes (*AUST2*, *MAPT*, *AGT* and *USH1C*) and two microRNAs (hsa-mir-100 and hsa-mir-378) were downregulated, all of which were associated with tumor malignancy. The putative microRNA-mRNA interactions identified in our analysis may play an important role in tumor progression.

## 3. Discussion

We conducted this study to investigate the long-term effects of hypoxia in 786-O, a VHL-defective renal cell carcinoma cell line, to identify potential genes and microRNAs associated with tumor malignancy using NGS and bioinformatics. Long-term hypoxia promoted the ability of colony formation and transwell migration compared to normoxia. In addition, some upregulated and downregulated genes in 786-O under long-term hypoxia were observed in clinical ccRCC samples along with malignant grade. These findings may provide a new direction for the study of potential molecular mechanisms associated with the progression of ccRCC.

Cancer progression is a long process and occurs in environments surrounding tumor cells which are deficient in nutrients and oxygen. This stressful environment can then cause the tumor cells to become more malignant as they adapt to the environment. We hypothesized that long-term hypoxia can partially mimic the stressful conditions where tumor cells grow. To test this hypothesis, we used a hypoxia workstation for long-term hypoxia to reduce exposure to normoxic conditions during the subculture procedure. We focused on the effects of hypoxia and so we did not change the serum concentration in the medium. We found that the 786-O cells that underwent long-term hypoxia exhibited increased colony formation and transwell migration ability, implying that long-term hypoxia induced more aggressive characteristics. However, we did not observe a significant change in wound healing assay (Figure S1), which indicated that the increased transwell migration ability in long-term hypoxia treated 786-O cells may be associated with elevated chemoattractive properties.

Hypoxia-induced HIF1/2α-dependent mechanisms have been widely reported to regulate gene expression. In 786-O cell line, we postulated that hypoxia treatment-affected gene expression is majorly through HIF1/2α-independent mechanisms. Hypoxia condition can cause increased production of reactive oxygen species (ROS) [20,21]. The accumulation of ROS is involved in activation of NF-κB-regulated signaling [22,23] and can also cause endoplasmic reticulum (ER) stress [24,25] and metabolic reprogramming [26,27] in cancer cells. In addition, chronic oxidative stress can induce chromosomal instability [28]. These hypoxia-induced mechanisms are associated with regulation of gene expression in response to stressful stimulations.

To elucidate these long-term effects of hypoxia in the 786-O cells, we also investigated short-term hypoxia. Through NGS analysis, we identified 42 upregulated genes and 18 downregulated genes in both short- and long-term hypoxia, suggesting that these genes may be regulated by hypoxia-specific mechanisms. Excluding these genes, 78 genes were upregulated and 60 genes downregulated in short-term hypoxia, compared to 279 genes and 193 genes, respectively, in long-term hypoxia. We therefore hypothesized that short-term hypoxia-specific genes may be rapidly regulated to adapt to stress in response to sudden exposure to hypoxia and that the genes associated with long-term hypoxia may be mostly regulated by the accumulation of reactive oxygen species (ROS) or genetic instability.

In short-term hypoxia, the differentially expressed genes were strongly associated with the TNF signaling pathway and the activation of TNF signaling via ROS has been demonstrated [29,30]. The upregulated genes, including *CSF2*, *PTGS2*, *SOCS3*, *CXCL3* and *CXCL2* are downstream products of TNF signaling. Colony stimulating factor 2 (CSF2) and prostaglandin-endoperoxide synthase 2 (PTGS2) were also observed in long-term hypoxia, suggesting hypoxia-specific regulation. The overexpression of CSF2 has been associated with poor survival in patients with urothelial carcinoma and NF-κB-induced CSF2 has been shown to promote tumor metastasis in patients with lung cancer and breast cancer [31,32]. *PTGS2*, also called *COX-2*, has been reported to play a critical role in tumor growth and metastasis in colon cancer [33,34] and high expressions of *CXCL2* and *CXCL3* have been associated with tumor metastasis [35,36,37]. *SOCS3* has also been reported to play a role in anti-proliferation and this may be regulated by negative feedback signaling [38,39]. The signaling axis of TNF-ROS-NF-κB induced by hypoxia [40] in tumor cells has also been reported to lead to the production of various inflammatory cytokines [41].

Various biological processes are strongly regulated by long-term hypoxia, including basal carcinoma, TNF signaling and cancer pathways. In TNF signaling, *CXCL1*, *IL6*, *CCL20* and *CCL5* have been shown to be upregulated and *VCAM1*, *IL18R1* and *MAPK13* downregulated. *CXCL1* is a critical cytokine involved in tumor metastasis, angiogenesis and chemoresistance and a high expression of *CXCL1* has been associated with a poor prognosis [42,43,44,45]. The oncogenic role of IL6 has also been well described [46,47]. Paracrine *CCL20* has been shown to induce epithelial-mesenchymal transition in breast cancer [48,49] and *CCL5* has been shown to promote vascular endothelial growth factor *VEGF*-induced angiogenesis [50]. In addition, depletion of *IL18R1*, an *IL18* receptor, has been shown to enhance tumor growth due to inhibitory recruitment of tumor-infiltrating lymphocytes [51]. Silencing of *MAPK13* has also been shown to promote cell growth in triple-negative breast cancer [52]. The expression of *VCAM1* on the cell surface has been shown to increase the survival of tumor cells by decreasing the number of infiltrating lymphocytes [53,54]. However, our results showed that the level of *VCAM1* was downregulated in the 786-O cells subjected to long-term hypoxia. It is important to understand how TNF signaling is affected by long-term hypoxia. One possible effect may be the accumulation of ROS, leading to activation of NF-κB. Biological processes involve complicated signaling networks, which may be influenced by changes in other signaling cascades. In addition to TNF signaling, many molecules involved in the wingless-type MMTV integration site family (*WNT*) signaling pathway were affected, including *WNT3*, *WNT5A*, *WNT5B*, *WNT7B* and *FZD8*. The functions of WNT signaling in cancer progression include cell proliferation, migration and tumor angiogenesis [55,56,57]. Moreover, matrix metalloproteinase-2 (*MMP-2*), an extracellular matrix protease which is known to play an important role in cell migration [58], was upregulated in the 786-O cells that underwent long-term hypoxia. Furthermore, the increased expression of vascular edothelial growth factor A (*VEGFA)*, which can regulate angiogenesis and vascular permeability [59], indicated the enhanced malignancy in the 786-O cells that underwent long-term hypoxia. Taken together, these findings suggest potential mechanisms that may be involved in malignancy induced by long-term hypoxia. However, further studies are needed to elucidate the activity of each signaling pathway.

With regards to the clinical analysis of ccRCC samples from microarrays of GSE66272 and GSE73731, the upregulation of *L1CAM* and *FBN1* and downregulation of *AUTS2*, *MAPT*, *AGT* and *USH1C* were observed along with an increase in tumor grade. Combined with survival curve analysis, higher expressions of *L1CAM* and *FBN1* and lower expressions of *AUTS2*, *MAPT*, *AGT* and *USH1C* were associated with poor survival outcomes. These findings imply that *L1CAM* and *FBN1* may potentially exhibit oncogenic functionality and that *AUTS2*, *MAPT*, *AGT* and *USH1C* may act as tumor suppressors in renal cancer.

*L1CAM*, L1 cell adhesion molecule, is a transmembrane protein which has been reported to promote tumor progression and metastasis in gastric cancer [60]. The role of *L1CAM* in invasive tumors has also been reported [61]. In endometrial cancer, the expression of *L1CAM* has been shown to be a predictor of poor survival and this has been shown to be associated with an advanced stage [62]. *FBN1*, fibrillin-1, is an extracellular matrix molecule which has been shown to promote ovarian cancer metastasis [63]. In addition, the identification of hypermethylated *FBN1* in stool samples has been used to detect colorectal cancer [64]. These reports support our findings that *L1CAM* and *FBN1* are upregulated in ccRCC along with a higher malignant grade and may also provide a new target for the development of therapies or diagnostic tools for renal cancer. Most reports studying *MAPT* (microtubule-associated protein tau) and *AUTS2* (autism susceptibility gene 2 protein) have focused on neural diseases and only a few studies have investigated their functions in cancer progression. Currently, it is only known that the expression of *MAPT* has been correlated with drug resistance in breast and gastric cancer [65,66]. *AUTS2* has also been shown to be a potential therapeutic target for pancreatic cancer with liver metastasis [67]. However, our findings suggest the potential tumor suppression function of *MAPT* and *AUTS2* in the progression of ccRCC. Thus, further studies are warranted to elucidate the role of *MAPT* and *AUTS2* in renal cancer. *AGT*, *O*^6^-alkylguanine-DNA alkyltransferase, is an important DNA repair protein [68] and many studies have reported that hypoxia can inhibit DNA repair to promote tumor malignancy and genetic instability [69,70,71,72]. This is consistent with our findings in that long-term hypoxia led to the downregulation of *AGT* in 786-O and that this effect was also observed in clinical ccRCC samples with an advanced grade. *USH1C*, USH1 protein network component harmonin, is a scaffold protein that has been associated with Usher syndrome type 1C [73]. However, no previous study has investigated its role in cancer progression and its role in ccRCC still needs to be elucidated.

In small RNA analysis, we found that the downregulation of miR-100 and miR-378 was correlated with metastatic ccRCC. In addition, the expression of hsa-mir-100-3p was lower in long-term hypoxia (2.2-fold change) compared to normoxia. However, hsa-mir-100-3p is not the major form of hsa-mir-100 and the expression of hsa-mir-100-5p was also lower in long-term hypoxia (1.89-fold change). Several studies have reported that miR-100 has a tumor suppressor effect in oral cancer [74] and prostate cancer [75] and the overexpression of hsa-mir-100 has been reported to suppress cell growth in esophageal squamous cancer [76] and non-small cell lung cancer (NSCLC) cells [77]. *GSE37989* has also been associated with the significant downregulation of hsa-mir-100 in metastatic ccRCC [78]. Another potential microRNA, hsa-mir-378, has also been sown to be downregulated in metastatic ccRCC identified from *GSE37989*. Our sequencing data showed that the expressions of most members of the hsa-mir-378 family were decreased in the 786-O cells under long-term hypoxia, including hsa-mir-378a-3p, hsa-mir-378a-5p, hsa-mir-378d and hsa-mir-378i. MicroRNA-378 has been reported to be a tumor suppressor in gastric [79], colon [80] and prostate cancer [81]. However, in liver cancer, the overexpression of hsa-mir-378 has been shown to promote cell migration [82], suggesting the potential tissue-specific effect of hsa-mir-378. Further studies are needed to elucidate the function of hsa-mir-378 in the progression of renal cancer. In this study, we also analyzed the putative microRNA-gene interactions through prediction databases and this may provide a new direction to study the progression of renal cancer.

## 4. Material and Methods

### 4.1. Cell Culture

The RCC cell line, 786-O (ATCC^®^ CRL1932™) was purchased from ATCC (American Type Culture Collection, Manassas, VA, USA). Cells were grown in Roswell Park Memorial Institute (RPMI) 1640 (Lonza, Walkersville, MD, USA), supplemented with 10% fetal bovine serum (Gibco-BRL, Grand Island, NY, USA) and 10,000 U penicillin/10 μg streptomycin/25 μg amphotericin B per mL (Lonza, Walkersville, MD, USA). Trypsin- ethylenediaminetetraacetic acid (EDTA) (0.05%, Gibco-BRL, Grand Island, NY, USA) was used for subcultures. For normoxia, the cells were incubated at 37 °C, with 20% O_2_ and 5% CO_2_. For hypoxia, the cells were incubated and passaged in a physiological oxygen workstations InvivO_2_ 400 (Baker Ruskinn, Sanford, ME, USA) at 37 °C, with 1% O_2_ and 5% CO_2_ for 3 months.

### 4.2. Colony Formation Assay

786-O cells with the same number of passages (at least 20 passages) grown in normoxic and hypoxic conditions were used for the assay. The cells were seeded onto 6-well plates at 1 × 10^2^ cells/well and incubated for 9 days. The colonies in each well were stained with 0.4% crystal violet in 100% ethanol and counted. The results were derived from at least three independent experiments.

### 4.3. Transwell Assay

786-O cells with the same number of passages (at least 20 passages) grown in normoxic and hypoxic conditions were used for assay. 4 × 10^4^ cells were suspended in 300 μL serum-free RPMI 1640 and poured into 24-well hanging inserts (0.8 μm, EMD Millipore Corporation, Billerica, MA, USA) and 500 μL complete RPMI 1640 (Lonza) was added to the lower wells. After 22 h, the insert membranes were stained with 0.2% crystal violet in 20% methanol. The cells on the inner side were wiped off with cotton swabs and the migrated cells on the outside membrane were counted. The results were derived from at least three independent experiments.

### 4.4. QRT-PCR

Cell lysates were homogenized and harvested with Trizol reagent (Invitrogen Life Technologies, Carlsbad, CA, USA). Total RNAs were extracted using Phenol:Chloroform:Isoamyl alcohol (25:24:1, *v*/*v*, Invitrogen Life Technologies) and isopropanol (Sigma-Aldrich, Saint Louis, MO, USA) according to the manufacturer’s instructions. Total RNA concentration was determined by O.D value 260 nm using PowerWave™ XS Microplate Spectrophotometer (BioTek Instruments, Inc., Winooski, VT, USA). RNAs (500 ng) were reverse transcribed into cDNAs using PrimeScriptTM RT reagent Kit (Takara Bio, Tokyo, Japan) according to the manufacturer’s instructions. For microRNA, RNAs (2 μg) were reverse transcribed into cDNAs using Mir-X™ miRNA First-Strand Synthesis kit (Clontech Laboratories, Inc., Foster, CA, USA). Real-time PCR was performed by using Fast SYBR^®^ Green Master Mix (Applied Biosystems, Foster, CA, USA) and StepOne real-time PCR machine (Applied Biosystems). The relative expression level was quantified by 2^−∆∆*C*t^. All results were performed with at least 3 independent experiments. qRT-PCR primers were *L1CAM*—forward 5′-CCATTGGTCCTGGAGTGCAT-3′ and reverse 5′-TGCAGGGTCTTGTTGTGGTT-3′; *FBN1*—5′-CCTATGCCGAGGTGGTGTTT-3′ and reverse 5′-TGTCGATACACGCGGAGATG-3′; *AUTS2*—5′-GCCAGCACCTCCCATGTTT-3′ and reverse 5′-GCAGCGACATCGATAGGGTT-3′; *AGT*—5′-GACCCCACCTTCATACCTGC-3′ and reverse 5′-GTTTTGCAGCGACTAGCACC-3′; *USH1C*—5′-CGGCTCCTACGCATCAAGAA-3′ and reverse 5′-GCCAGGGTGTAGTCTGTCAC-3′; *MAPT*—5′-ATGCACCAAGACCAAGAGGG-3′ and reverse 5′-CCGCTGTTGGAGTGCTCTTA-3′. The microRNA primers (hsa-mir-100-3p, hsa-mir-100-5p, hsa-mir-378a-3p, hsa-mir-378a-5p, hsa-mir-378d and hsa-mir-378i) were ordered as custom oligonucleotide synthesis (Bio-search, Inc., San Rafael, CA, USA).

### 4.5. Next-Generation Sequencing (NGS)

Total RNA lysates were harvested using Trizol^®^ Reagent (Invitrogen Life Technologies) according to the manufacturer’s instructions. The expression profiles of protein-coding mRNA and microRNAs were evaluated by performing RNA-seq and small RNA-seq respectively [83]. The quality of OD260 nm was detected using an ND-1000 spectrophotometer (Nanodrop Technology, Wilmington, CA, USA). Samples were sent to Welgene Biotechnology Company (Taipei, Taiwan) for RNA preparation and sequencing analysis. The detailed information was provided in Supplementary information file.

### 4.6. Gene Expression Omnibus (GEO) Database Analysis

The GEO database includes a comprehensive amount of publicly submitted data, including microarrays, chips, RNA-seq and small RNA-seq of NGS data (Available online: https://www.ncbi.nlm.nih.gov/geo/, accessed on 8 August 2017 and 23 November 2017) [84]. In this project, we selected microarrays GSE66272 [85], GSE73731 [86] and GSE37989 [78] for further analysis. The gene expression levels were extracted using the GEO2R online tool (Available online: https://www.ncbi.nlm.nih.gov/geo/geo2r/, accessed on 8 August 2017 and 23 November 2017) and re-plotted using GraphPad Prism 5.0 software (GraphPad Software, La Jolla, CA, USA).

### 4.7. SurvExpress Analysis

SurvExpress integrates TCGA datasets (Available online: https://tcga-data.nci.nih.gov, accessed on 21 August 2017) of different cancer types to provide correlation analysis of gene expressions and survival outcomes. Survival curves comparing two populations of patients at high and low risk of renal cancer were plotted using the SurvExpress online database (Available online: http://bioinformatica.mty.itesm.mx/SurvExpress, accessed on 21 August 2017). The samples of each datasets were split into two risk groups with the same size, which was determined using the ordered prognostic index (PI, high value for high risk) [87]. The raw data were extracted and re-plotted using GraphPad Prism 5.0 software. The PI is the linear component of a Cox model, calculated as the gene expression value multiplied by values estimated from Cox fitting [88].

### 4.8. miRMap Database Analysis

The miRMap database was used to predict the microRNA target (Available online: http://mirmap.ezlab.org/, accessed on 20 July 2017) [89]. The putative targets were identified based on calculating the complementary ability of microRNA-mRNA interactions. The predictor also estimated the strength of mRNA repression to rank potential candidate targets using features including thermodynamics, evolution, probability and sequence-based features. The prediction results provided a list of putative target genes with miRmap scores, which were used as predictive reference values. The threshold of microRNA target prediction was set at a miRmap score ≥97.0 in this study.

### 4.9. DAVID Database Analysis

We used the Database for Annotation, Visualization and Integrated Discovery (DAVID, Available online: https://david.ncifcrf.gov/, accessed on 14 September 2017) combined with functional annotation databases including Gene Ontology (GO), Biological Process and KEGG Pathway [19] to classify genetic functions and analyze the related pathways. The list of genes was classified into clusters of related biological functions, signaling pathways and diseases by calculating the similarity of global annotation profiles using an agglomeration algorithm. It also provided the EASE score for analysis, which is a modified Fisher’s exact *p*-value. The reference score represented how specifically the user genes were involved in the category (for example: signaling pathways). We selected an EASE (a modified Fisher Exact *p*-value) score ≥1 to extend the clustering range in our analysis.

### 4.10. Gene Set Enrichment Analysis (GSEA)

GSEA (Available online: http://software.broadinstitute.org/gsea/index.jsp, accessed on 13 September 2017) is software used to compare two sets of gene expression data and determine which group has upregulated biological processes or pathways [90].

### 4.11. Statistical Analysis

The raw data extracted from the GEO database were statistically analyzed using the unpaired *t*-test to analyze two groups or one-way ANOVA with Tukey’s *post hoc* test to compare all pairs of columns in multiple group analysis using GraphPad Prism 5.0 software. 

## 5. Conclusions

The results showed that long-term hypoxia promoted the ability of colony formation and transwell migration compared to normoxia. In addition, the differentially expressed genes induced by long-term hypoxia were involved in various biological processes including cell proliferation, the TNF-signaling pathway, basal cell carcinoma and cancer pathways. The expressions of upregulated and downregulated genes were correlated with survival outcomes in patients with renal cancer. Besides, this study identified potential genes and microRNAs that may be regulated by long-term hypoxia. These factors may be novel targets to study the molecular mechanisms involved in the advanced stage of renal cancer.

## Figures and Tables

**Table 1:** KEGG pathway analysis of differentially expressed genes in 786-O treated with short-term and long-term hypoxia.

Condition	KEGG Pathway	Counts	*p*-Value	Up Genes	Down Genes	Fold Enrichment
Short-term hypoxia	TNF signaling pathway	5	0.024267	*CSF2*, *PTGS2*, *SOCS3*, *CXCL3*, *CXCL2*	-	4.46497803
Long-term hypoxia	Basal cell carcinoma	8	0.000622	*FZD8*, *WNT7B*, *TCF7*, *WNT3*, *WNT5B*	*WNT5A*, *PTCH1*, *GLI1*	5.374818
	TNF signaling pathway	9	0.007555	*CXCL1*, *CSF2*, *IL6*, *PTGS2*, *CCL20*, *CCL5*	*VCAM1*, *IL18R1*, *MAPK13*	3.137423
	Pathways in cancer	19	0.018474	*JUP*, *FZD8*, *WNT7B*, *IL6*, *TCF7*, *WNT3*, *WNT5B*, *PTGS2*, *PGF*, *VEGFA*, *EGLN3*, *GNG4*, *MMP2*	*WNT5A*, *PDGFRB*, *PTCH1*, *FGF12*, *MMP1*, *GLI1*	1.786477
	Proteoglycans in cancer	12	0.018829	*FZD8*, *WNT7B*, *WNT3*, *WNT5B*, *ANK3*, *VEGFA*, *MMP2*	*WNT5A*, *MAPK13*, *HPSE*, *LUM*, *PTCH1*	2.217112
	Glycerolipid metabolism	6	0.019391	*ALDH2*, *DGKI*	*DGKA*, *DGAT1*, *AKR1B10*, *PNPLA3*	3.822607

KEGG—Kyoto Encyclopedia of Genes and Genomes; TNF—tumor necrosis factor pathway.

**Table 2:** Detailed information of Gene set enrichment analysis (GSEA) analysis.

Normoxia vs. Long-Term_Hypoxia (Class A vs. Class B)						
GSEA Set Name	MSigDB	Counts	ES	NES	NOM *p*-Value	FDR *q*-Value
REGULATION_OF_CELL_PROLIFERATION	C5	15	−0.39	−1.64	0.021	0.241
CELL_PROLIFERATION_GO_0008283	C5	22	−0.33	−1.61	0.047	0.215

The criteria were a NOM (nominal) *p*-value < 0.05 and a false discovery rate (FDR) *q*-value < 0.25. Platform—MSigDB, C5, gene ontology (GO) gene sets; ES—enrichment score; NES—normalized enrichment score.

**Table 3:** The target prediction of downregulated microRNAs in 786-O under long-term hypoxia treatment.

miRNA ID	Gene Symbol	miRmap	TargetScan	miRDB	miRNA ID	Gene Symbol	miRmap	TargetScan	miRDB
hsa-mir-100-3p	*CEP97*	+	+	-	hsa-mir-939-5p	*DGKI*	+	+	+
hsa-mir-125b-2-3p	*PYGO1*	+	+	-	hsa-mir-939-5p	*OAS1*	+	-	-
hsa-mir-1306-5p	*STRA6*	+	-	-	hsa-mir-939-5p	*CAMK1D*	+	-	-
hsa-mir-1306-5p	*CDC42BPG*	+	-	-	hsa-mir-939-5p	*KCNK3*	+	+	-
hsa-mir-1306-5p	*PAQR7*	+	-	+	hsa-mir-939-5p	*HMGA1*	+	+	-
hsa-mir-1306-5p	*PGM2L1*	+	+	-	hsa-mir-939-5p	*AFAP1*	+	+	-
hsa-mir-1306-5p	*GPRC5B*	+	-	-	hsa-mir-939-5p	*OLFML2A*	+	+	-
hsa-mir-1306-5p	*EGLN3*	+	-	-	hsa-mir-939-5p	*CHRDL1*	+	+	-
hsa-mir-148a-5p	*EIF4E3*	+	+	-	hsa-mir-939-5p	*ALPK3*	+	+	+
hsa-mir-149-5p	*MDGA1*	+	-	-	hsa-mir-939-5p	*LIMD2*	+	+	-
hsa-mir-149-5p	*RAB3IL1*	+	+	+	hsa-mir-939-5p	*CRABP2*	+	+	-
hsa-mir-149-5p	*GNG4*	+	-	-	hsa-mir-939-5p	*TCF7*	+	+	+
hsa-mir-149-5p	*TANC2*	+	-	-	hsa-mir-939-5p	*GPRC5B*	+	+	-
hsa-mir-188-5p	*GDAP1*	+	-	+	hsa-mir-939-5p	*INHBB*	+	+	-
hsa-mir-193a-3p	*OLFML2A*	+	-	-	hsa-mir-939-5p	*SLC7A6*	+	+	-
hsa-mir-193a-3p	*SLC5A3*	+	-	-	hsa-mir-939-5p	*TIMP2*	+	+	+
hsa-mir-218-5p	*MDGA1*	+	+	+	hsa-mir-939-5p	*VEGFA*	+	+	+
hsa-mir-218-5p	*LOX*	+	-	+	hsa-mir-9-5p	*FBN1*	+	+	+
hsa-mir-218-5p	*LRIG1*	+	+	+	hsa-mir-9-5p	*EIF4E3*	+	+	-
hsa-mir-3200-3p	*CDH13*	+	+	+	hsa-mir-9-5p	*SPTLC2*	+	+	+
hsa-mir-378a-3p	*OTUB2*	+	+	+	hsa-mir-6511b-5p	*DIRAS2*	+	+	-
hsa-mir-378a-3p	*ALPK3*	+	+	-	hsa-mir-6511b-5p	*MDGA1*	+	+	+
hsa-mir-378a-5p	*PLXNA2*	+	+	+	hsa-mir-6511b-5p	*APLN*	+	+	-
hsa-mir-378d	*OTUB2*	+	+	+	hsa-mir-6511b-5p	*KCNK3*	+	+	-
hsa-mir-378d	*ALPK3*	+	+	-	hsa-mir-6511b-5p	*AFAP1*	+	+	-
hsa-mir-378i	*OTUB2*	+	+	+	hsa-mir-6511b-5p	*SPNS3*	+	+	+
hsa-mir-378i	*ALPK3*	+	+	-	hsa-mir-6511b-5p	*GNG4*	+	+	+
hsa-mir-5094	*EPB41L4B*	+	-	+	hsa-mir-6511b-5p	*WNT7B*	+	+	-
hsa-mir-5094	*SPOCK1*	+	+	-	hsa-mir-6511b-5p	*KIAA0513*	+	+	+
hsa-mir-873-3p	*WNT3*	+	+	+	hsa-mir-6511b-5p	*SPTLC2*	+	+	-
hsa-mir-939-5p	*CYP26B1*	+	+	+					

The potential microRNA-mRNA interactions identified through miRmap were analyzed by using TargetScan and miRDB. MicroRNAs in table are downregulated with fold change ≤−2 and protein-coding mRNAs are upregulated with fold change >2. “+”and “-”mean predicted and not predicted respectively.

**Table 4:** The target prediction of upregulated microRNAs in 786-O under long-term hypoxia treatment.

miRNA ID	Gene Symbol	miRmap	TargetScan	miRDB
*hsa-mir-122-5p*	*CYBRD1*	+	-	+
*hsa-mir-195-5p*	*IL17RE*	+	+	-
*hsa-mir-195-5p*	*LIN7A*	+	+	-
*hsa-mir-195-5p*	*PLSCR4*	+	+	+
*hsa-mir-195-5p*	*SEMA3A*	+	+	+
*hsa-mir-195-5p*	*MOCS1*	+	+	-
*hsa-mir-195-5p*	*KSR1*	+	+	-
*hsa-mir-195-5p*	*PDK4*	+	+	+
*hsa-mir-195-5p*	*BTG2*	+	+	+
*hsa-mir-195-5p*	*CLDN2*	+	+	+
*hsa-mir-195-5p*	*PTCH1*	+	+	-
*hsa-mir-195-5p*	*SYNJ1*	+	+	+
*hsa-mir-195-5p*	*CACNA2D1*	+	+	-
*hsa-mir-204-5p*	*SAMD12*	+	+	-
*hsa-mir-204-5p*	*MAPT*	+	-	-
*hsa-mir-204-5p*	*EGR1*	+	-	+
*hsa-mir-381-3p*	*ULK2*	+	-	+
*hsa-mir-381-3p*	*CACNA2D1*	+	-	-
*hsa-mir-411-5p*	*LUM*	+	-	-
*hsa-mir-486-5p*	*NTRK3*	+	-	-
*hsa-mir-767-5p*	*VASH1*	+	+	-
*hsa-mir-943*	*PLSCR4*	+	+	-

The potential microRNA-mRNA interactions identified through miRmap were analyzed by using TargetScan and miRDB. MicroRNAs in table are upregulated with fold change ≥2 and protein-coding mRNAs are downregulated with fold change <−2. “+”and “-”mean predicted and not predicted respectively.

## Supplementary Materials

Supplementary materials can be found at www.mdpi.com/1422-0067/18/12/2657/s1.

## Abbreviations

RCC	renal cell carcinoma
ccRCC	clear cell RCC
HIF	hypoxia-inducible factor
VHL	von Hippel-Lindau
FPKM	fragments per kilobase of transcript per million mapped reads
GSEA	gene set enrichment analysis
ROS	reactive oxygen species
CSF2	colony stimulating factor 2
PTGS2	prostaglandin-endoperoxide synthase 2
MAPT	microtubule-associated protein tau
AUTS2	autism susceptibility gene 2 protein
