# Supplementary material for: Systematic Analysis of Transcriptomic Profile of Renal Cell Carcinoma under Long-Term Hypoxia Using Next-Generation Sequencing and Bioinformatics
Source: Int J Mol Sci. 2017 Dec 7;18(12):2657. doi: 10.3390/ijms18122657 (PMC5751259; doi:10.3390/ijms18122657)
Supplement: Supplementary file 1 [file ijms-18-02657-s001.pdf]

Supplement information

1. Next-generation sequencing (NGS)

The expression profiles of protein-coding mRNA and microRNAs were evaluated by performing RNA-seq and small RNA-seq respectively. Total RNA was extracted by using Trizol® Reagent (Invitrogen, USA) according to manufacturer's instructions. The quality of OD260 nm was detected by using a ND-1000 spectrophotometer (Nanodrop Technology, USA). Samples were applied to Welgene Biotechnology Company (Welgene, Taipei, Taiwan) for RNA preparation and sequencing analysis. RNA samples were first qualitated by using a Bioanalyzer 2100 (Agilent Technology, USA) with RNA 6000 labchip kit (Agilent Technologies, USA).

For small RNA-seq, to construct the small RNA library and perform deep sequencing, samples were prepared using Illumina sample preparation kit according to the TruSeq Small RNA Sample Preparation Guide. The total RNA was ligated with 3′ and 5′ adaptors and reverse-transcribed into cDNA by PCR amplification. The harvested cDNA constructs were fractionated by size on a 6% polyacrylamide gel electrophoresis and the bands containing the 18-40 nucleotide RNA fragments (140-155 nucleotide in length with both adapters) were purified. Libraries were then sequenced on an Illumina GAIIx instrument (50 cycle single read) and the sequencing results were processed with the Illumina software. To analyze small RNA sequiencing, the sequences were applied to go through a filtering process to obtain qualified reads. ConDeTri was used to trim or remove the reads according to the quality score. The qualified reads were then analyzed using miRDeep2 to clip the 3′ adapter sequence and remove shorter reads (< 18 nucleotides), before aligning reads to the human genome from UCSC. Because miRNAs are usually mapped to few genomic locations, only reads mapped perfectly to the genome five or less times were used for miRNA detection. MiRDeep2 was used to estimate expression levels of miRNAs. The criteria for microRNAs selection are fold change ≥ 2, and reads per million (RPM) ≥ 1.

For RNA-seq, all procedures were carried out according to the manufacture's protocol from Illumina. Library construction of all samples were used by Agilent's SureSelect Strand Specific RNA Library Preparation Kit for 75SE (Single-End or Paired-End) sequencing on Solexa platform. The sequence was directly determined using sequencing-by-synthesis technology via the TruSeq SBS Kit. Raw sequences were obtained from the Illumina Pipeline software bcl2fastq v2.0 and expected to generate 30M (million reads or Gb) per sample. For RNA-seq analysis, initially, the sequences generated went through a filtering process to obtain qualified reads. Trimmomaticwas implemented to trim or remove the reads according to the quality score. Qualified reads after filtering low-quality data were analyzed using TopHat/Cufflinks [1] for gene expression estimation. The gene expression level was calculated as FPKM (Fragments Per Kilobase of transcript per Million mapped reads). For differential expression analysis, CummeRbund was employed to perform statistical analyses of gene expression profiles. The reference genome and gene annotations were retrieved from Ensembl database.

The information of material & method was provided from Welgene Biotech Company, Taipei, Taiwan.

2. Wound healing assay

Cells were seeded 2 x 10^5 cells / 12 well and incubated at 37°C for 24h. A wound line was scratched across cell layer by using 200μl tip and the medium was changed with 0.5% FBS RPMI. The pictures of 0h and 20h were captured by 40x (4x*10x) microscopy. The open area (no cells) was analyzed by using TScratch software and the migration area was determined as ΔMigration Area (%) = 100*(open area (0h)-open area (20h)). Mean±SEM and P-value were calculated by unpaired t-test with GraphPad Prism 5 software.

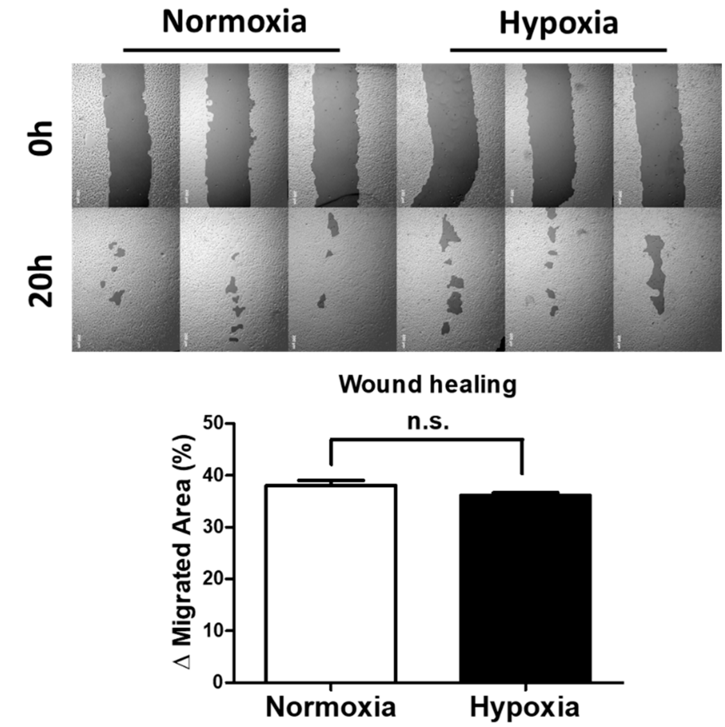

Supplementary Figure 1. Analysis of cell migration in 786-O cells treated with normoxia and long-term hypoxia by wound healing assay. Wound healing assay was performed in 768-O cells treated with normoxia and long-term hypoxia. The open area (no cells) was analyzed by using TScratch software and the migration area was determined as ΔMigration Area (%) = 100*(open area (0h)-open area (20h)). The scale bar = 200μm. Mean±SEM and P-value were calculated by unpaired t-test with GraphPad Prism 5 software. We didn't observe the significant difference of ΔMigration Area (%) comparing 786-O treated with normoxia and long-term hypoxia.

Reference

1. Trapnell, C.; Roberts, A.; Goff, L.; Pertea, G.; Kim, D.; Kelley, D.R.; Pimentel, H.; Salzberg, S.L.; Rinn, J.L.; Pachter, L. Differential gene and transcript expression analysis of RNA-seq experiments with TopHat and Cufflinks. *Nat. Prot*. **2012**, *7*, 562-578.
